# Supplementary material for: Mapping the evolution of fertility support policies in China: A content and instrumental analysis
Source: PLoS One. 2025 Oct 9;20(10):e0332137. doi: 10.1371/journal.pone.0332137 (PMC12510515; doi:10.1371/journal.pone.0332137)
Supplement: S1 Appendix — (ZIP) [file pone.0332137.s001.zip › S1 Appendix. 226 original policy documents/153-中华人民共和国家庭教育促进法(FBM-CLI-1-5077898).doc]

中华人民共和国家庭教育促进法

**发布部门：** [全国人大常委会](https://www.pkulaw.com/law/chl?Aggs.IssueDepartment=201)

**发文字号：**中华人民共和国主席令第98号

**发布日期：**2021.10.23

**实施日期：**2022.01.01

**时效性：** [现行有效](https://www.pkulaw.com/law/chl?Aggs.TimelinessDic=01)

**效力级别：** [法律](https://www.pkulaw.com/law/chl?Aggs.EffectivenessDic=XA0101)

**法规类别：** [教育综合规定](https://www.pkulaw.com/law/chl?Aggs.Category=09101)

中华人民共和国主席令
（第九十八号）

　　《中华人民共和国家庭教育促进法》已由中华人民共和国第十三届全国人民代表大会常务委员会第三十一次会议于2021年10月23日通过，现予公布，自2022年1月1日起施行。

中华人民共和国主席　习近平
　　2021年10月23日

中华人民共和国家庭教育促进法
（2021年10月23日第十三届全国人民代表大会常务委员会第三十一次会议通过）
目　　录

　　第一章　总　　则
　　第二章　家庭责任
　　第三章　国家支持
　　第四章　社会协同
　　第五章　法律责任
　　第六章　附　　则

第一章　总　　则

　　**第一条**　为了发扬中华民族重视家庭教育的优良传统，引导全社会注重家庭、家教、家风，增进家庭幸福与社会和谐，培养德智体美劳全面发展的社会主义建设者和接班人，制定本法。

　　**第二条**　本法所称家庭教育，是指父母或者其他监护人为促进未成年人全面健康成长，对其实施的道德品质、身体素质、生活技能、文化修养、行为习惯等方面的培育、引导和影响。

　　**第三条**　家庭教育以立德树人为根本任务，培育和践行社会主义核心价值观，弘扬中华民族优秀传统文化、革命文化、社会主义先进文化，促进未成年人健康成长。

　　**第四条**　未成年人的父母或者其他监护人负责实施家庭教育。

　　国家和社会为家庭教育提供指导、支持和服务。

　　国家工作人员应当带头树立良好家风，履行家庭教育责任。

　　**第五条**　家庭教育应当符合以下要求：

　　（一）尊重未成年人身心发展规律和个体差异；

　　（二）尊重未成年人人格尊严，保护未成年人隐私权和个人信息，保障未成年人合法权益；

　　（三）遵循家庭教育特点，贯彻科学的家庭教育理念和方法；

　　（四）家庭教育、学校教育、社会教育紧密结合、协调一致；

　　（五）结合实际情况采取灵活多样的措施。

　　**第六条**　各级人民政府指导家庭教育工作，建立健全家庭学校社会协同育人机制。县级以上人民政府负责妇女儿童工作的机构，组织、协调、指导、督促有关部门做好家庭教育工作。

　　教育行政部门、妇女联合会统筹协调社会资源，协同推进覆盖城乡的家庭教育指导服务体系建设，并按照职责分工承担家庭教育工作的日常事务。

　　县级以上精神文明建设部门和县级以上人民政府公安、民政、司法行政、人力资源和社会保障、文化和旅游、卫生健康、市场监督管理、广播电视、体育、新闻出版、网信等有关部门在各自的职责范围内做好家庭教育工作。

　　**第七条**　县级以上人民政府应当制定家庭教育工作专项规划，将家庭教育指导服务纳入城乡公共服务体系和政府购买服务目录，将相关经费列入财政预算，鼓励和支持以政府购买服务的方式提供家庭教育指导。

　　**第八条**　人民法院、人民检察院发挥职能作用，配合同级人民政府及其有关部门建立家庭教育工作联动机制，共同做好家庭教育工作。

　　**第九条**　工会、共产主义青年团、残疾人联合会、科学技术协会、关心下一代工作委员会以及居民委员会、村民委员会等应当结合自身工作，积极开展家庭教育工作，为家庭教育提供社会支持。

　　**第十条**　国家鼓励和支持企业事业单位、社会组织及个人依法开展公益性家庭教育服务活动。

　　**第十一条**　国家鼓励开展家庭教育研究，鼓励高等学校开设家庭教育专业课程，支持师范院校和有条件的高等学校加强家庭教育学科建设，培养家庭教育服务专业人才，开展家庭教育服务人员培训。

　　**第十二条**　国家鼓励和支持自然人、法人和非法人组织为家庭教育事业进行捐赠或者提供志愿服务，对符合条件的，依法给予税收优惠。

　　国家对在家庭教育工作中做出突出贡献的组织和个人，按照有关规定给予表彰、奖励。

　　**第十三条**　每年5月15日国际家庭日所在周为全国家庭教育宣传周。

第二章　家庭责任

　　**第十四条**　父母或者其他监护人应当树立家庭是第一个课堂、家长是第一任老师的责任意识，承担对未成年人实施家庭教育的主体责任，用正确思想、方法和行为教育未成年人养成良好思想、品行和习惯。

　　共同生活的具有完全民事行为能力的其他家庭成员应当协助和配合未成年人的父母或者其他监护人实施家庭教育。

　　**第十五条**　未成年人的父母或者其他监护人及其他家庭成员应当注重家庭建设，培育积极健康的家庭文化，树立和传承优良家风，弘扬中华民族家庭美德，共同构建文明、和睦的家庭关系，为未成年人健康成长营造良好的家庭环境。

　　**第十六条**　未成年人的父母或者其他监护人应当针对不同年龄段未成年人的身心发展特点，以下列内容为指引，开展家庭教育：

　　（一）教育未成年人爱党、爱国、爱人民、爱集体、爱社会主义，树立维护国家统一的观念，铸牢中华民族共同体意识，培养家国情怀；

　　（二）教育未成年人崇德向善、尊老爱幼、热爱家庭、勤俭节约、团结互助、诚信友爱、遵纪守法，培养其良好社会公德、家庭美德、个人品德意识和法治意识；

　　（三）帮助未成年人树立正确的成才观，引导其培养广泛兴趣爱好、健康审美追求和良好学习习惯，增强科学探索精神、创新意识和能力；

　　（四）保证未成年人营养均衡、科学运动、睡眠充足、身心愉悦，引导其养成良好生活习惯和行为习惯，促进其身心健康发展；

　　（五）关注未成年人心理健康，教导其珍爱生命，对其进行交通出行、健康上网和防欺凌、防溺水、防诈骗、防拐卖、防性侵等方面的安全知识教育，帮助其掌握安全知识和技能，增强其自我保护的意识和能力；

　　（六）帮助未成年人树立正确的劳动观念，参加力所能及的劳动，提高生活自理能力和独立生活能力，养成吃苦耐劳的优秀品格和热爱劳动的良好习惯。

　　**第十七条**　未成年人的父母或者其他监护人实施家庭教育，应当关注未成年人的生理、心理、智力发展状况，尊重其参与相关家庭事务和发表意见的权利，合理运用以下方式方法：

　　（一）亲自养育，加强亲子陪伴；

　　（二）共同参与，发挥父母双方的作用；

　　（三）相机而教，寓教于日常生活之中；

　　（四）潜移默化，言传与身教相结合；

　　（五）严慈相济，关心爱护与严格要求并重；

　　（六）尊重差异，根据年龄和个性特点进行科学引导；

　　（七）平等交流，予以尊重、理解和鼓励；

　　（八）相互促进，父母与子女共同成长；

　　（九）其他有益于未成年人全面发展、健康成长的方式方法。

　　**第十八条**　未成年人的父母或者其他监护人应当树立正确的家庭教育理念，自觉学习家庭教育知识，在孕期和未成年人进入婴幼儿照护服务机构、幼儿园、中小学校等重要时段进行有针对性的学习，掌握科学的家庭教育方法，提高家庭教育的能力。

　　**第十九条**　未成年人的父母或者其他监护人应当与中小学校、幼儿园、婴幼儿照护服务机构、社区密切配合，积极参加其提供的公益性家庭教育指导和实践活动，共同促进未成年人健康成长。

　　**第二十条**　未成年人的父母分居或者离异的，应当相互配合履行家庭教育责任，任何一方不得拒绝或者怠于履行；除法律另有规定外，不得阻碍另一方实施家庭教育。

　　**第二十一条**　未成年人的父母或者其他监护人依法委托他人代为照护未成年人的，应当与被委托人、未成年人保持联系，定期了解未成年人学习、生活情况和心理状况，与被委托人共同履行家庭教育责任。

　　**第二十二条**　未成年人的父母或者其他监护人应当合理安排未成年人学习、休息、娱乐和体育锻炼的时间，避免加重未成年人学习负担，预防未成年人沉迷网络。

　　**第二十三条**　未成年人的父母或者其他监护人不得因性别、身体状况、智力等歧视未成年人，不得实施家庭暴力，不得胁迫、引诱、教唆、纵容、利用未成年人从事违反法律法规和社会公德的活动。

第三章　国家支持

　　**第二十四条**　国务院应当组织有关部门制定、修订并及时颁布全国家庭教育指导大纲。

　　省级人民政府或者有条件的设区的市级人民政府应当组织有关部门编写或者采用适合当地实际的家庭教育指导读本，制定相应的家庭教育指导服务工作规范和评估规范。

　　**第二十五条**　省级以上人民政府应当组织有关部门统筹建设家庭教育信息化共享服务平台，开设公益性网上家长学校和网络课程，开通服务热线，提供线上家庭教育指导服务。

　　**第二十六条**　县级以上地方人民政府应当加强监督管理，减轻义务教育阶段学生作业负担和校外培训负担，畅通学校家庭沟通渠道，推进学校教育和家庭教育相互配合。

　　**第二十七条**　县级以上地方人民政府及有关部门组织建立家庭教育指导服务专业队伍，加强对专业人员的培养，鼓励社会工作者、志愿者参与家庭教育指导服务工作。

　　**第二十八条**　县级以上地方人民政府可以结合当地实际情况和需要，通过多种途径和方式确定家庭教育指导机构。

　　家庭教育指导机构对辖区内社区家长学校、学校家长学校及其他家庭教育指导服务站点进行指导，同时开展家庭教育研究、服务人员队伍建设和培训、公共服务产品研发。

　　**第二十九条**　家庭教育指导机构应当及时向有需求的家庭提供服务。

　　对于父母或者其他监护人履行家庭教育责任存在一定困难的家庭，家庭教育指导机构应当根据具体情况，与相关部门协作配合，提供有针对性的服务。

　　**第三十条**　设区的市、县、乡级人民政府应当结合当地实际采取措施，对留守未成年人和困境未成年人家庭建档立卡，提供生活帮扶、创业就业支持等关爱服务，为留守未成年人和困境未成年人的父母或者其他监护人实施家庭教育创造条件。

　　教育行政部门、妇女联合会应当采取有针对性的措施，为留守未成年人和困境未成年人的父母或者其他监护人实施家庭教育提供服务，引导其积极关注未成年人身心健康状况、加强亲情关爱。

　　**第三十一条**　家庭教育指导机构开展家庭教育指导服务活动，不得组织或者变相组织营利性教育培训。

　　**第三十二条**　婚姻登记机构和收养登记机构应当通过现场咨询辅导、播放宣传教育片等形式，向办理婚姻登记、收养登记的当事人宣传家庭教育知识，提供家庭教育指导。

　　**第三十三条**　儿童福利机构、未成年人救助保护机构应当对本机构安排的寄养家庭、接受救助保护的未成年人的父母或者其他监护人提供家庭教育指导。

　　**第三十四条**　人民法院在审理离婚案件时，应当对有未成年子女的夫妻双方提供家庭教育指导。

　　**第三十五条**　妇女联合会发挥妇女在弘扬中华民族家庭美德、树立良好家风等方面的独特作用，宣传普及家庭教育知识，通过家庭教育指导机构、社区家长学校、文明家庭建设等多种渠道组织开展家庭教育实践活动，提供家庭教育指导服务。

　　**第三十六条**　自然人、法人和非法人组织可以依法设立非营利性家庭教育服务机构。

　　县级以上地方人民政府及有关部门可以采取政府补贴、奖励激励、购买服务等扶持措施，培育家庭教育服务机构。

　　教育、民政、卫生健康、市场监督管理等有关部门应当在各自职责范围内，依法对家庭教育服务机构及从业人员进行指导和监督。

　　**第三十七条**　国家机关、企业事业单位、群团组织、社会组织应当将家风建设纳入单位文化建设，支持职工参加相关的家庭教育服务活动。

　　文明城市、文明村镇、文明单位、文明社区、文明校园和文明家庭等创建活动，应当将家庭教育情况作为重要内容。

第四章　社会协同

　　**第三十八条**　居民委员会、村民委员会可以依托城乡社区公共服务设施，设立社区家长学校等家庭教育指导服务站点，配合家庭教育指导机构组织面向居民、村民的家庭教育知识宣传，为未成年人的父母或者其他监护人提供家庭教育指导服务。

　　**第三十九条**　中小学校、幼儿园应当将家庭教育指导服务纳入工作计划，作为教师业务培训的内容。

　　**第四十条**　中小学校、幼儿园可以采取建立家长学校等方式，针对不同年龄段未成年人的特点，定期组织公益性家庭教育指导服务和实践活动，并及时联系、督促未成年人的父母或者其他监护人参加。

　　**第四十一条**　中小学校、幼儿园应当根据家长的需求，邀请有关人员传授家庭教育理念、知识和方法，组织开展家庭教育指导服务和实践活动，促进家庭与学校共同教育。

　　**第四十二条**　具备条件的中小学校、幼儿园应当在教育行政部门的指导下，为家庭教育指导服务站点开展公益性家庭教育指导服务活动提供支持。

　　**第四十三条**　中小学校发现未成年学生严重违反校规校纪的，应当及时制止、管教，告知其父母或者其他监护人，并为其父母或者其他监护人提供有针对性的家庭教育指导服务；发现未成年学生有不良行为或者严重不良行为的，按照有关法律规定处理。

　　**第四十四条**　婴幼儿照护服务机构、早期教育服务机构应当为未成年人的父母或者其他监护人提供科学养育指导等家庭教育指导服务。

　　**第四十五条**　医疗保健机构在开展婚前保健、孕产期保健、儿童保健、预防接种等服务时，应当对有关成年人、未成年人的父母或者其他监护人开展科学养育知识和婴幼儿早期发展的宣传和指导。

　　**第四十六条**　图书馆、博物馆、文化馆、纪念馆、美术馆、科技馆、体育场馆、青少年宫、儿童活动中心等公共文化服务机构和爱国主义教育基地每年应当定期开展公益性家庭教育宣传、家庭教育指导服务和实践活动，开发家庭教育类公共文化服务产品。

　　广播、电视、报刊、互联网等新闻媒体应当宣传正确的家庭教育知识，传播科学的家庭教育理念和方法，营造重视家庭教育的良好社会氛围。

　　**第四十七条**　家庭教育服务机构应当加强自律管理，制定家庭教育服务规范，组织从业人员培训，提高从业人员的业务素质和能力。

第五章　法律责任

　　**第四十八条**　未成年人住所地的居民委员会、村民委员会、妇女联合会，未成年人的父母或者其他监护人所在单位，以及中小学校、幼儿园等有关密切接触未成年人的单位，发现父母或者其他监护人拒绝、怠于履行家庭教育责任，或者非法阻碍其他监护人实施家庭教育的，应当予以批评教育、劝诫制止，必要时督促其接受家庭教育指导。

　　未成年人的父母或者其他监护人依法委托他人代为照护未成年人，有关单位发现被委托人不依法履行家庭教育责任的，适用前款规定。

　　**第四十九条**　公安机关、人民检察院、人民法院在办理案件过程中，发现未成年人存在严重不良行为或者实施犯罪行为，或者未成年人的父母或者其他监护人不正确实施家庭教育侵害未成年人合法权益的，根据情况对父母或者其他监护人予以训诫，并可以责令其接受家庭教育指导。

　　**第五十条**　负有家庭教育工作职责的政府部门、机构有下列情形之一的，由其上级机关或者主管单位责令限期改正；情节严重的，对直接负责的主管人员和其他直接责任人员依法予以处分：

　　（一）不履行家庭教育工作职责；

　　（二）截留、挤占、挪用或者虚报、冒领家庭教育工作经费；

　　（三）其他滥用职权、玩忽职守或者徇私舞弊的情形。

　　**第五十一条**　家庭教育指导机构、中小学校、幼儿园、婴幼儿照护服务机构、早期教育服务机构违反本法规定，不履行或者不正确履行家庭教育指导服务职责的，由主管部门责令限期改正；情节严重的，对直接负责的主管人员和其他直接责任人员依法予以处分。

　　**第五十二条**　家庭教育服务机构有下列情形之一的，由主管部门责令限期改正；拒不改正或者情节严重的，由主管部门责令停业整顿、吊销营业执照或者撤销登记：

　　（一）未依法办理设立手续；

　　（二）从事超出许可业务范围的行为或作虚假、引人误解宣传，产生不良后果；

　　（三）侵犯未成年人及其父母或者其他监护人合法权益。

　　**第五十三条**　未成年人的父母或者其他监护人在家庭教育过程中对未成年人实施家庭暴力的，依照《[中华人民共和国未成年人保护法](https://www.pkulaw.com/chl/883cecd7fe38636dbdfb.html)》、《[中华人民共和国反家庭暴力法](https://www.pkulaw.com/chl/ac57ba1df4413457bdfb.html)》等法律的规定追究法律责任。

　　**第五十四条**　违反本法规定，构成违反治安管理行为的，由公安机关依法予以治安管理处罚；构成犯罪的，依法追究刑事责任。

第六章　附　　则

　　**第五十五条**　本法自2022年1月1日起施行。

© 北大法宝：（[www.pkulaw.com](https://www.pkulaw.com/)) 专业提供法律信息、法学知识和法律软件领域各类解决方案。北大法宝为您提供丰富的参考资料，正式引用法规条文时请与标准文本核对。
欢迎查看所有[产品和服务](https://www.pkulaw.com/helps/2.html)。法宝快讯：[如何快速找到您需要的检索结果？](https://www.pkulaw.com/helps/70.html)[法宝](https://www.pkulaw.com/helps/69.html)V6有何新特色？

扫描二维码阅读原文

**原文链接：**<https://www.pkulaw.com/chl/99c5277887339564bdfb.html>
